# Hydroxyl ion diffusion through intact and resorbed roots at various time intervals

**Published:** 2011-08-15

**Authors:** Nasrin Roghanizad, Fatemeh Bahreini, Mehdi Vatanpour

**Affiliations:** 1 *Department of Endodontics, Dental Branch, Islamic Azad University of Medical Sciences, Tehran, Iran*; 2 *General Dentist, Dental Research Center, Shahid Beheshti University of Medical Sciences, Tehran, Iran*

**Keywords:** Calcium hydroxide, Diffusion, Hydroxyl ion, PH, Root resorption

## Abstract

**INTRODUCTION:** The purpose of this study was to investigate root surface pH changes over a period of 4 weeks following treatment with calcium-hydroxide dressing of intact and resorbed roots.

**MATERIALS & METHODS:** The canals of 30 single-rooted teeth were instrumented with ProTaper rotary files and randomly divided into 2 experimental groups of 14 each, and one control group with two samples. To simulate external resorption in group 2 and control, two defects were made on the buccal and lingual mid-root surfaces of each tooth with a diamond bur (1×1mm). Teeth were then submerged in 20% sulfuric acid for 4 days. Canals of 14 intact teeth in group 1, and 14 resorbed teeth in group 2 were filled with calcium hydroxide Ca(OH)_2_ while canals of resorbed teeth in control group were filled with saline. All teeth were then placed in a glass vial containing 15mL of deionized water (pH≈7). The pH of water was measured at 0 and 1, 4, 7, 10, 14, 21 and 28 days using pH microelectrode. Independent t-test and repeated measured ANOVA analyzed the data.

**RESULTS:** Except a mild decline at 7days and 14days, the average pH values showed increased during the time periods of this study. Day 28 showed the maximum pH average value in both groups (7.67-7.72) (P<0.05). Significant increase of pH values were detected at different time intervals compared with the baseline time (P<0.05).

**CONCLUSION:** Diffusion of hydroxyl ions was similar in both resorbed and intact roots. In both groups pH values were significantly higher than the baseline pH at the various times.

## INTRODUCTION

**C**alcium hydroxide (Ca(OH)_2_) has been the most commonly used intracanal medicament for managing resorption in traumatic teeth (1-3); diffusion of hydroxyl ions through dentinal tubules changes the pH on the external surface of the resorbed roots often arresting resorption (4).

Following the placement of Ca(OH)_2_ in the canal, hydroxyl ions diffuse through dentinal tubules potentially increasing pH up to 8-10. This alkaline environment in areas of resorption can interfere with osteoclastic activity and inhibit inflammatory root resorption (2). Removal of intracanal smear layer with 10mL of 17% EDTA before Ca(OH)_2_ placement will help ions diffusion through dentinal tubules (5). Esberard *et al.* claimed that following usage of Ca(OH)_2_ in the canals a high pH was maintained at the resorbed root surface for at least 120 days (6). Saif *et al.* showed that hydroxyl ions diffusion through dentinal tubules decreased during the first 7 days after Ca(OH)_2_ placement in both intact and resorbed roots (7).

External root resorption is a destructive process that may continue even after primary stability. As these lesions cannot be repaired with dentin and cementum, perforation of root canal to the external environment and microbial micro-leakage or even fracture of the tooth in advanced root resorption is expected. Therefore the time periods of Ca(OH)_2_ usage for control and treatment of external root resorption is very critical. However, sufficient information is lacking regarding the curve of pH values in different periods of time. The purpose of this *ex vivo *study was to investigate hydroxyl ions diffusion over a period of 4 weeks following Ca(OH)_2_ dressing in intact and resorbed roots. 

## MATERIALS AND METHODS

After a pilot study, 30 extracted permanent human premolars were used in this experimental study. The teeth were stored in 0.9% sodium chloride (4,7). Presence of a single root canal, absence of calcification, internal and external root resorption was verified with radiographs (7). Crowns were amputated at the cementoenamel junction with a #557 carbide bur (Henry Schein, Melville, NY). 

External surfaces of all roots were covered with Parafilm strips (Parafilm M, Laboratory Film, Chicago, USA) to avoid contamination with NaOCl during canal irrigation (4). A K-file #10 (Dentsply, Maillefer, USA) was placed in the canal 1mm over the apical foramen as patency file and then 1.5mm was subtracted to establish working length (5). All roots were prepared with a crown-down technique using ProTaper (Dentsply, Maillefer, USA) rotary instruments to #40 master apical file. The smear layer in all the canals were removed with a final 3 mL 17% EDTA 1-minute irrigation followed by 10mL 5.25% NaOCl (7).

The samples were randomly divided into two experimental groups of 14 each and one control group of two samples using random number table (Group 1: intact roots, Group 2: simulated resorbed roots).

In group 2 and control group samples, two defects were made on the buccal and lingual mid-root surface with a #801 size 010 diamond round bur (1×1mm) (Tees Kavan Co., Tehran, Iran). After placing 3mm of Coltosol, (Coltène, Altstätten, Switzerland) over a small cotton pellet on coronal cavities, samples were submerged in melted rose wax. The wax over the created defects was cleaned with a small heated instrument. Subsequently, waxed samples were submerged in 20% sulfuric acid for 4 days to simulate external root resorption (8). 

After 4 days, the wax was washed under boiling water. Steam jet (SC590 multi steamer steam cleaner, *Kenwood,* Australia) was then used for final cleansing. To neutralize the effect of sulfuric acid, samples of group 2 were stored in normal saline for 24 hours. Root surfaces were rinsed under 10mL of deionized water (7). After drying the canals with paper points, calcium hydroxide (Dentsply Herpo, Petrópolis, RJ, Brazil) was prepared with normal saline according to manufacturer’s instruction and placed in all experimental canals using lentulo spiral #35 (Mani, Tochigi-ken, Japan) (7). Canals in the control group were rinsed with normal saline.

All coronal access cavities were sealed with a small cotton pellet and 3mm of Coltosol. The external coronal and apical 3mm of all roots were sealed with heated sticky wax (7).

The teeth were then placed in a glass vial containing 15mL of deionized water (pH≈7) (7). The pH of each vial was measured at 0 and 1, 4, 7, 10, 14, 21 and 28 days using a pH micro-electrode (Portable pH meter Hi8314, Hanna, Italy) which was calibrated to pH values of 4, 7 and 9 at each time period. The electrode was kept in each vial for 2 minutes before reading the pH (according to manufacturer’s instruction). After each of the measurements, the electrode was rinsed with deionized water and dried.

Independent t-test was used to compare the average pH of the two groups at each time interval. For comparing the different time periods of each group, repeated measured ANOVA was applied. For all statistical test type 1 error was set at α=0.05 and expected power of analysis was 0.8.

## RESULTS

The two groups had significant difference only at day 14 (independent t-test, P=0.006).

**Figure 1:**
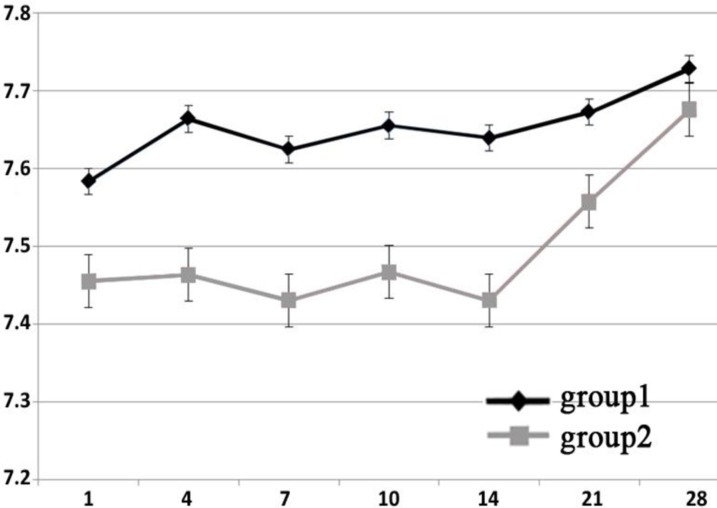
pH of external environment of two groups at the different time intervals

The mean pH values in each group had significant differences at the various time periods (repeated measured ANOVA, P<0.0001). Multiple comparison test showed significant differences in each group until the 4^th^ day (Tukey test, P<0.05) after which no significant difference was seen between time periods in each group (Tukey HSD, P>0.05). There was no interaction between time and groups (P>0.05).

The pH values increased over time (Figure 1). In each group, the pH value of different time periods had significant difference with baseline (Time=0) (Tukey HSD, P<0.05).

## DISCUSSION

Except a mild decline on day 7 and 14, the average pH values showed an increasing pattern throughout the study. Day 28 showed the maximum average pH value in both experimental groups. 

Since pH values can be affected by NaOCl, the root surfaces were covered with parafilm strips to prevent contamination of NaOCl during canal irrigation (4).

Several studies have used burs to simulate external root resorption (4-7,9,10). However, the smear layer blocks dentinal tubules. Even after a final flush with deionized water, using EDTA for removing smear layer can affect the pH of root surface (7). Some studies had an upper baseline pH due to smear layer blockage (4,6,9).

Additionally, burs create regular resorption patterns in dentin, whereas *in vivo *resorption follows no certain pattern. Therefore, after creating the regular equivalent defects all samples were submerged in sulfuric acid to simulate irregular resorption pattern (8). To prevent any affect of Sulfuric acid on pH, subsequent to removing the waxes with boiled water and steam jet, roots were placed in saline for 24 hours. Finally the roots were rinsed with 10mL deionized water (7).

The average pH values increased during our study in both groups, concurring with Esberard *et al.’s *study (6). They showed continuous hydroxyl ions diffusion over 120 days. Also according to Nerwich *et al.’s* study the pH of the cervical outer dentin increased within two weeks and reached a constant level after three weeks (4). In our study, pH of external root surface reached a significant level at day 4 and despite increase of pH, there was no significant release of hydroxyl ion.

However, Saif *et al.* (7) demonstrated decreas-ing pH value until day 5 after which it remained unchanged.

This difference can be explained as follows: they did not have two groups (intact *vs*. resorbed) running at the same time. In fact they used the same samples after 30 days to create defect and did not even change the canals’ Ca(OH)_2_ content.

The present study is the only study which has two separate groups (intact *vs.* resorbed) running at the same time and pH recordings at regular frequent time periods.

Maximum average of pH value in present study was 7.72 in intact and 6.67 in resorbed group. The maximum average of pH value in our and Saif *et al.* study (7) are lower than others (4,6,9). This is probably because other researchers used electrodes directly in the defect to measure pH, while we and Saif *et al.* measured the pH of surrounding water of the root. Moreover, the defect diameter in our and Saif *et al.* (7) studies were less than the others (1mm vs. 1.5-1.75mm). It means that we exposed less tubules and therefore it seems logical to have lower pH.

According to this ex vivo study, hydroxyl ion diffusion was significant up to day 4. The diffusion rates of hydroxyl ions in both intact and resorbed roots were similar and no significant difference was observed.

## CONCLUSION

The increasing pattern of pH with the progress of time in both groups confirms long term use of Ca(OH)_2_ as an inhibitory agent against destructive inflammation caused by traumatic injuries. Further studies are required because of dissimilar reactions of Ca(OH)_2_ in different conditions.
